# Admitted Dengue Cases among the Adult Dengue Positive Cases in a Tertiary Care Centre: A Descriptive Cross-sectional Study

**DOI:** 10.31729/jnma.7675

**Published:** 2022-09-30

**Authors:** Durga Dhungana, Bidhya Banstola, Mahesh Banjara

**Affiliations:** 1 Department of Internal Medicine, Manipal College of Medical Sciences, Pokhara, Kaski, Nepal; 2 Department of Nursing Administration, Pokhara Academy of Health Sciences, Pokhara, Kaski, Nepal; 3 Department of Laboratory Medicine, Gandaki Medical College Teaching Hospital and Research Centre, Pokhara, Kaski, Nepal

**Keywords:** *dengue*, *fever*, *headache*, *leukopenia*, *thrombocytopenia*

## Abstract

**Introduction:**

Dengue is an infectious disease. This disease is prevalent mainly in the terai belts of Nepal. But in the last few years, the cases are in increasing trend in the hilly areas of Nepal. The aim of this study was to find out the prevalence of admitted dengue cases among adult dengue-positive cases in a tertiary care centre.

**Methods:**

This was a descriptive cross-sectional study done in a tertiary teaching hospital after obtaining ethical approval from the Institutional Review Committee (Reference number: 063/2077/2078). Convenience sampling was used. The data of serologically confirmed dengue cases, during the period of 1 August 2019 to 1 December 2019, of ages above 15 years, were collected from the hospital records. Point estimate and 95% Confidence Interval were calculated.

**Results:**

Out of 922 adult dengue-positive patients, 347 (37.63%) (36.04-39.22, 95% Confidence Interval) were admitted. Among them, 154 (44.38%) cases were seen during the month of September. A total of 264 (76.08%) were the inhabitants of the Kaski district. A total of one hundred seventy eight (51.29%) cases were males. The most common symptoms seen were fever among 335 (96.54%) patients and headache among 141 (40.63%) patients. Leukopenia was seen in 192 (55.33%) patients and thrombocytopenia was seen in 165 (47.55%) of the admitted cases.

**Conclusions:**

The prevalence of admitted dengue cases was higher as compared to other studies done in similar settings.

## INTRODUCTION

Dengue is an emerging infectious disease in Nepal. Transmission and the increasing global burden of disease are being fuelled by population growth, urbanization, increasingly favourable ecologic and environmental habitats for Aedes mosquitoes compounded by poor vector control, and the ease of international travel.^1^

The first reported case of dengue was in 2004 A.D. from Chitwan. From 2006-19 AD, the total number of cases were 8391.^2^ In regards to Gandaki Province, the cases were nil till the last few years with one case reported each in the year 2073 B.S and 2074 B.S. A total of 2679 cases were reported in 2019. The majority of cases were reported from the Kaski district.^3^ Research on dengue has been limited which mainly focused on Terai belts and demographic characteristics.^4,5^ This study focuses on all aspects including the laboratory parameters.

The aim of this study was to find out the prevalence of admitted dengue cases among adult dengue-positive cases in a tertiary care centre.

## METHODS

This was a descriptive cross-sectional study done among dengue patients, diagnosed serologically with positive NS1 antigen and/or IgM antibody tests, which were admitted to the medicine ward or had visited the Out Patient Department (OPD) of the medicine department of Gandaki Medical College Teaching Hospital and Research Center during the period of 1 August 2019 to 1 December 2019 from hospital records after obtaining approval from the Institutional Review Committee of Gandaki Medical College (Reference number: 063/2077/2078). Convenience sampling was done. The sample size was calculated using the formula:


$$\begin{array}{ll} \text{n} & ={\text{Z}}^{2}\times \frac{\text{p}\times \text{q}}{{\text{e}}^{\text{2}}}\\ & =1.96^{2}\times \frac{0.5\times 0.5}{0.05^{2}}\\ & =385 \end{array}$$

Where,

n= minimum required sample size
Z= 1.96 at 95% Confidence Interval (CI)
p= prevalence taken as 50% for maximum sample size calculation
q= 1-p
e= margin of error, 5%

Hence, the minimum required sample size was 385. As a convenience sampling technique was used, the sample size was doubled and a sample size of 770 was obtained. However, a sample size of 922 was taken for the study.

Patient details were obtained from the medical records department for the admitted cases and the laboratory for the out-patient cases. Data was collected with respect to the demographic profile of the patients, and laboratory diagnostic methods with the haematological and biochemical investigations done. Proforma included data on age, sex, address, presenting symptoms, signs, area of admission, duration of hospital stay (for admitted cases), outcome (death or discharged) and laboratory tests. Among admitted cases, all of them had undergone a complete blood count. Dengue was diagnosed based on the NS1 antigen test or IgM antibody test. However, other laboratories and radiological tests were done on a needed basis as seen in data from the medical records section. Laboratory tests included aspartate transaminase (AST), alanine transaminase (ALT) and renal function tests (RFT). Radiological tests included X-ray and ultrasonography. Thrombocytopenia was defined as a platelet count of less than 150000/mm^3^ of blood. Leukopenia was defined as a leukocyte count of less than 4000/mm^3^ of blood. Cases of dengue were classified as per the WHO classification of dengue.^6^

Data was entered into Microsoft Excel 2017 and checked for repetition. Data regarding outpatient cases were incomplete in regards to haematological and biochemical profiles hence were included in the demographic analysis only. Data of admitted cases were only analyzed in regard to laboratory data. A total of 922 cases were obtained after removing repeated cases matching the address, gender and age. Data were entered and analyzed in IBM SPSS Statistics 20.0. Point estimate and 95% CI were calculated.

## RESULTS

Out of 922 adult dengue-positive patients, 347 (37.63%) (36.04-39.22, 95% CI) were admitted. Among them, 154 (44.38%) cases were seen during the month of September. A total of 264 (76.08%) were from the Kaski district (Table 1).

**Table 1:** Socio-demographic characteristics (n= 347).

Characteristics		n (%)
**Month of admission**	August	108 (31.12)
	September	154 (44.38)
	October	58 (16.72)
	November	27 (7.78)
**Gender**	Male	178 (51.30)
	Female	169 (48.70)
**Age (in years)**	16 to 30	201 (57.93)
	31 to 45	98 (28.24)
	46 to 60	32 (9.22)
	61 and above	16 (4.61)
**District**	Kaski	264 (76.08)
	Tanahun	61 (17.58)
	Others	22 (6.34)

Three (0.86%) cases were managed at the ICU. A total of 38 (10.95%) had some known comorbidities. The majority i.e. 16 (4.61%) of them were hypertensive (Table 2).

**Table 2:** Clinical characteristics (n= 347).

Characteristics		n (%)
**Comorbidities**	Yes	38 (10.95)
	Hypertension	16 (4.61)
	Diabetes	8 (2.31)
	Pregnancy	4 (1.15)
	Others	13 (3.74)
**Lab diagnosis**	NS1 antigen positive	337 (97.12)
	IgM antibody positive	5 (1.44)
	Both NS1 and IgM positive	5 (1.44)
**Admission**	ICU	3 (0.86)
	Ward	344 (99.14)
**Dengue category**	Dengue without warning signs	252 (72.62)
	Dengue with warning signs	95 (27.38)
Hospital stay duration	≤4 days	291 (83.86)
	>4 days	56 (16.14)

Fever was present in 335 (96.54%) admitted cases with the gastrointestinal system involved in 104 (29.97%) (Table 3).

**Table 3:** Presenting symptoms among admitted cases (n= 347).

Symptoms	n (%)
Fever	335 (96.54)
Headache	141 (40.63)
Nausea or vomiting	80 (23.05)
Diarrhoea	13 (3.75)
Abdominal pain	11 (3.17)
Myalgia	66 (19.02)
Retro-orbital pain	23 (6.63)
Rash	16 (4.61)
Bleeding	11 (3.17)

Leukopenia in 192 (55.30%) and thrombocytopenia in 165 (47.60%) were common findings.

## DISCUSSION

This data on the outbreak of dengue in Pokhara found that about 37.6% of the dengue patients got admitted for treatment. Males were slightly more affected than females (M:F=1.1:1). This finding was similar to the study done in Nepal, India, Taiwan and Malaysia.^7-11^ The median age of presentation was 29 years. The older age group represented a minority (<5%) of cases. These findings are similar to the study done in tertiary teaching hospitals in Malaysia and Bangladesh during the months of July to September 2019.^9,12^

Most of the admitted cases were managed at the hospital ward level with only three cases needing intensive care and one case expired during the course of treatment. Hypertension (42%) and diabetes mellitus (21%) were the most common comorbidities present. This was similar to the findings of other studies.^12,13^ Fever (96.5%) was the most common clinical symptom in this study and was a common finding in other studies too.^8,9,11-20^ The second most common symptom was headache (40.6%) which was similar to the findings of other studies.^8,13^ Proportion of patients with headache and myalgia was less in this study as compared to the studies.^9,12,17,19,21^ This might be due to the entry of the main symptom only during admission and ignoring the less troublesome symptoms by the patient in history taking.

Leukopenia and thrombocytopenia were seen respectively in 55.3% and 47.6% of total admitted cases. Similar findings were seen in a study conducted in a hospital in eastern India.^14^ Percentage of patients with findings of leukopenia and thrombocytopenia are variable due to the variation in the cut-off values taken in comparison with other studies. With similar cut-off values also, the difference may be likely due to the timing of blood analysis also. Some may have been in the febrile phase whereas others may have been in the critical phase.^12,13,18,19,22^

With the newer classification of dengue by WHO, the cases with warning signs were 95 (27.3%). This was less as compared to the study done in Bangladesh where 45.5% of admitted cases were with warning signs.^12^ This difference is probably due to the parameters taken into account. Radiological findings and detailed clinical examination were not present as well as findings that could have developed later haven't been taken into account. However, a study done in India showed only 11% of cases with warning signs.^20^ This variation is probably due to the study in the latter part being done among both admitted and OPD cases; however, this and the former study were conducted among only admitted cases.

The limitations of the study are that it is a single-centre study and it is of retrospective nature. The serological type of dengue wasn't investigated. The differentiation between primary and secondary dengue was not done. Also, the haematological profile of the outpatient cases couldn't be included in the full analysis. However, the major power of this study is the large sample size.

## CONCLUSIONS

The prevalence of admitted dengue cases was higher as compared to other studies done in similar settings. Fever is the most common symptom of dengue in adult patients. With the increasing cases in hilly areas of Nepal, community-level programs are needed to be implemented for a timely decrease in morbidity and mortality due to dengue. Leukopenia and thrombocytopenia are common findings in dengue patients and should be regarded as suggestive factors.
